# Evaluation of Fife Forensic CMHT Liaison Services Over 10 Years

**DOI:** 10.1192/bjo.2022.80

**Published:** 2022-06-20

**Authors:** Leanne Duthie, Elspeth Pike

**Affiliations:** NHS Fife, Fife, United Kingdom

## Abstract

**Aims:**

Fife FCMHT offer two forms of liaison; a court liaison service and a consultation service open to any professional requiring guidance on managing a person with mental disorder and offending behaviour. Our aims are to evaluate these services by analysing the number of referrals, reason for referral and outcomes in order to assess how our services are being used and help identify any areas for improvement.

**Methods:**

Details about each referral made to the court liaison and consultation services and outcomes were recorded from January 2011 to December 2021. Data were analysed in excel.

**Results:**

***Court Liaison Service***

1044 referrals were made; 778 of these were assessed. 98.7% were seen on day of referral. 76 required inpatient admission, 9 of whom had to be remanded in custody to await appropriate bed. Age ranged 15–78 years. Of those deemed fit to continue through court, 33% were felt to require further mental health input.

***Consultation Service***

280 referrals were made. Age ranged 15–83 years. The majority of referrals to this service came from criminal justice social work and NHS fife services. The majority of referrals were for specific advice or help with risk assessment and management. The average time between referral and consultation was 9.4 days.

**Conclusion:**

Reassuringly, our team responds promptly to referrals.

25.5% of referrals made to the court service did not require assessment after triage. Only 7.3% of referrals required diversion away from the court system. Whilst 33% of those deemed fit to continue were identified as requiring further mental health input, this was often in the form of signposting to local services. As referrals are usually seen by health care in custody, this suggests mental health training for these teams would be of benefit to prevent delays in court proceedings and prevent unnecessary referrals.

Of concern are those patients remanded in custody to await a psychiatric bed. Whilst numbers are small, it is an unacceptable outcome for these patients. This occurs due to no bed being available or a requirement for assessment by the admitting unit. This mirrors findings from the Barron Report.

Our consultation service sees requests from a vast array of professionals. We believe this to be an efficient way for services to access the expertise within our team, avoiding unnecessary referrals causing delays to patient care. The majority of these referrals were for advice over a specific matter which can be dealt with succinctly by the team.

